# Phylogenomic Reconstruction of the Oomycete Phylogeny Derived from 37 Genomes

**DOI:** 10.1128/mSphere.00095-17

**Published:** 2017-04-12

**Authors:** Charley G. P. McCarthy, David A. Fitzpatrick

**Affiliations:** Department of Biology, Genome Evolution Laboratory, Maynooth University, Maynooth, Co. Kildare, Ireland; Carnegie Mellon University

**Keywords:** oomycete, phylogeny, *Phytophthora*, species phylogeny, phylogenomics, supermatrix, supertrees

## Abstract

The oomycetes are a class of microscopic, filamentous eukaryotes within the *Stramenopiles-Alveolata-Rhizaria* (SAR) supergroup which includes ecologically significant animal and plant pathogens, most infamously the causative agent of potato blight *Phytophthora infestans*. Single-gene and concatenated phylogenetic studies both of individual oomycete genera and of members of the larger class have resulted in conflicting conclusions concerning species phylogenies within the oomycetes, particularly for the large *Phytophthora* genus. Genome-scale phylogenetic studies have successfully resolved many eukaryotic relationships by using supertree methods, which combine large numbers of potentially disparate trees to determine evolutionary relationships that cannot be inferred from individual phylogenies alone. With a sufficient amount of genomic data now available, we have undertaken the first whole-genome phylogenetic analysis of the oomycetes using data from 37 oomycete species and 6 SAR species. In our analysis, we used established supertree methods to generate phylogenies from 8,355 homologous oomycete and SAR gene families and have complemented those analyses with both phylogenomic network and concatenated supermatrix analyses. Our results show that a genome-scale approach to oomycete phylogeny resolves oomycete classes and individual clades within the problematic *Phytophthora* genus. Support for the resolution of the inferred relationships between individual *Phytophthora* clades varies depending on the methodology used. Our analysis represents an important first step in large-scale phylogenomic analysis of the oomycetes.

**IMPORTANCE** The oomycetes are a class of eukaryotes and include ecologically significant animal and plant pathogens. Single-gene and multigene phylogenetic studies of individual oomycete genera and of members of the larger classes have resulted in conflicting conclusions concerning interspecies relationships among these species, particularly for the *Phytophthora* genus. The onset of next-generation sequencing techniques now means that a wealth of oomycete genomic data is available. For the first time, we have used genome-scale phylogenetic methods to resolve oomycete phylogenetic relationships. We used supertree methods to generate single-gene and multigene species phylogenies. Overall, our supertree analyses utilized phylogenetic data from 8,355 oomycete gene families. We have also complemented our analyses with superalignment phylogenies derived from 131 single-copy ubiquitous gene families. Our results show that a genome-scale approach to oomycete phylogeny resolves oomycete classes and clades. Our analysis represents an important first step in large-scale phylogenomic analysis of the oomycetes.

## Abstract (precis)

The oomycetes are a class of eukaryotes and include ecologically significant animal and plant pathogens. Single-gene and multigene phylogenetic studies of individual oomycete genera and of members of the larger classes have resulted in conflicting conclusions concerning interspecies relationships among these species, particularly for the *Phytophthora* genus. The onset of next-generation sequencing techniques now means that a wealth of oomycete genomic data is available. For the first time, we have used genome-scale phylogenetic methods to resolve oomycete phylogenetic relationships. We used supertree methods to generate single-gene and multigene species phylogenies. Overall, our supertree analyses utilized phylogenetic data from 8,355 oomycete gene families. We have also complemented our analyses with superalignment phylogenies derived from 131 single-copy ubiquitous gene families. Our results show that a genome-scale approach to oomycete phylogeny resolves oomycete classes and clades. Our analysis represents an important first step in large-scale phylogenomic analysis of the oomycetes.

## INTRODUCTION

**FIG 1:**
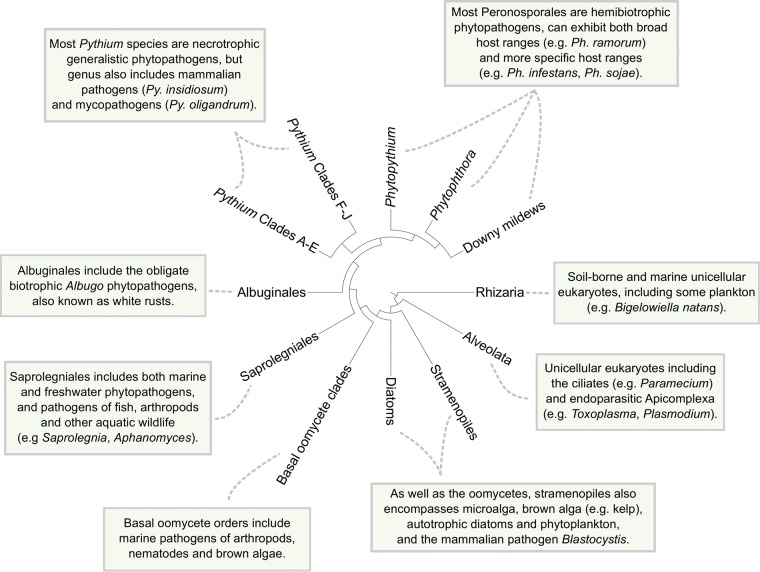
Consensus phylogeny of the oomycetes class within the greater SAR grouping, including information pertaining to various taxa. The cladogram was adapted from Judelson (10).

The oomycetes are a class of microscopic eukaryotes which include some of the most ecologically destructive marine and terrestrial eukaryotic species (1). Oomycete species display filamentous morphology and ecological roles very similar to those of fungi and were historically regarded as a basal fungal lineage (2). As morphological and molecular studies have improved since the latter half of the 20th century, the oomycetes have come to be understood as very distant relations of “true” fungi. They have independently evolved similar morphology and lifestyles through convergent evolution and limited interkingdom horizontal gene transfer (HGT) (2–5). Present phylogenomic studies place the oomycetes in the diverse stramenopiles lineage within the *Stramenopiles-Alveolata-Rhizaria* (SAR) eukaryotic supergroup (6–10) (Fig. 1). The stramenopiles were previously placed within *Chromista* (11) and then within the “chromalveolates” supergroup (*Chromista* plus *Alveolata*) on the basis of a hypothesized last common ancestor on the plastid lineage (12, 13). While early phylogenetic analyses supported the concept of a single origin for the “chromalveolate” plastid (14, 15), later plastome-wide and nuclear phylogenetic and HGT analyses have consistently failed to support a monophyletic chromalevolate grouping (16–21). In contrast, molecular evidence for the monophyly of the current SAR supergroup has been demonstrated in multiple phylogenetic analyses (18, 20, 22–26).

The oomycetes are thought to have diverged from diatoms between the Late Proterozoic and the mid-Paleozoic eras (~0.4 to 0.6 billion years ago [bya]) (27, 28) and have been found to have been present as early as the Devonian period (~400 million years ago [mya]) in the fossil record (29). Though many described species are phytopathogens, oomycete phytopathogenicity is thought to be a derived trait which has evolved independently in many lineages (30). Many species are as yet unsampled, and the class phylogeny of the oomycetes is still subject to revision; with current data, however, the oomycetes can be split into the earliest diverging clades and the later “crown” taxa (31–33) (Fig. 1). With the exception of some species infecting terrestrial nematodes (31), the earliest diverging oomycete clades are otherwise exclusively marine in habitat (1). The remaining “crown” oomycetes can be subdivided into the predominantly marine and freshwater “saprolegnian” branches and the predominantly terrestrial “peronosporalean” branches, which diverged in the Early Mesozoic era (1, 28, 34–36). The “saprolegnian” branches include the fish pathogen *Saprolegnia*, also known as “cotton mould” (37), and the animal- and plant-pathogenic *Aphanomyces* genus (34, 38). The “peronosporalean” branches include the best-characterized oomycete taxa, *Phytophthora* and *Pythium*, and the more basal *Albuginales* order (1, 35). The majority of “peronosporalean” oomycetes are phytopathogens, although *Pythium* includes species capable of infecting animals or acting as mycoparasitic biocontrol agents (39, 40) (Fig. 1).

**FIG 2:**
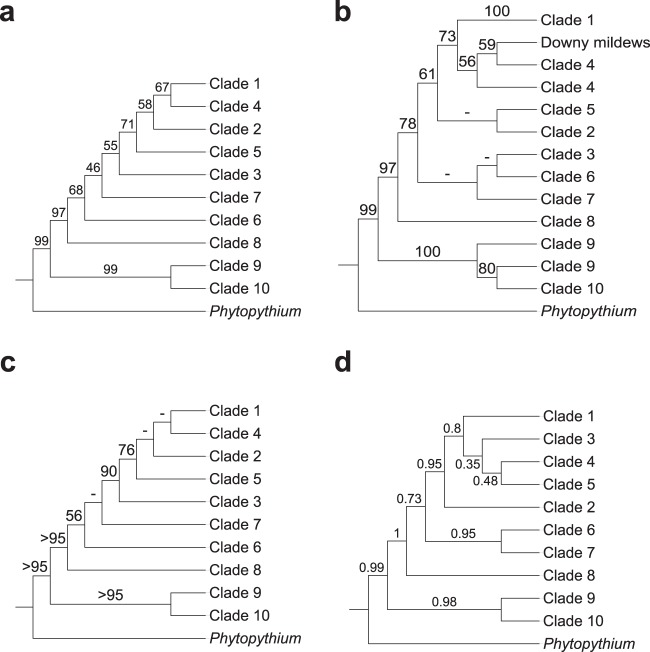
Congruence of the *Peronosporales* order among recent multilocus phylogenetic analyses. (a) Seven-locus maximum-likelihood (ML)/maximum-parsimony (MP)/Bayesian phylogeny of *Phytophthora* by Blair et al. (42). (b) Minimum-evolution (ME)/ML/Bayesian phylogeny of *Phytophthora* and downy mildews by Runge et al. (46). (c) Eleven-locus ML/MP/Bayesian phylogeny of *Phytophthora* by Martin et al. (47). (d) Six-locus coalescent phylogeny of *Phytophthora* by Martin et al. (47). Support values, where given, represent maximum-likelihood bootstrap support, except for panel d, where Bayesian posterior probabilities are given instead.

*Phytophthora* is the largest genus (>120 described species) within the order *Peronosporales* and was divided into 10 phylogenetic clades on the basis of initial internal transcribed spacer (ITS) analysis and, later, combined nuclear and mitochondrial analyses (41, 42) (Fig. 2a). The largest clades (clades 1, 2, 7, and 8) are further divided into subclades, while the smallest clades (clades 5 and 10) contain fewer than five described species at present (43, 44). Initial ITS phylogeny data reported by Cooke et al. (41) suggested that *Phytophthora* was paraphyletic with respect to basal clades 9 and 10; however, later multigene and combined nuclear and mitochondrial studies have placed these clades within *Phytophthora* (42, 44, 45). Generally, species within *Phytophthora* clades do not share consistent morphological features or reproductive strategies, although clades 6 to 8 form a distinct branch of terrestrial species with predominantly nonpapillate sporangia within the genus tree (44). While many recent phylogenetic analyses have supported the current designation by Blair et al. (42) of 10 distinct phylogenetic clades within *Phytophthora*, many of the same analyses draw conflicting conclusions as to the relationships among these clades. In their analysis, Blair et al. (42) found strong support by maximum-likelihood, maximum-parsimony, and Bayesian methods for the 10 phylogenetic clades using data from seven highly conserved nuclear loci (including markers from 28S ribosomal DNA [rDNA], Hsp90, and β-tubulin) from 82 *Phytophthora* species (Fig. 2a). The relationship between the clades reported in Blair et al. (42) was mostly upheld in a follow-up analysis by Runge et al. (46) which included homologous data from an additional 39 *Phytophthora* species and other *Peronosporales* species. One noticeable difference was that their analysis placed clades 3, 6, and 7 as sister clades within a monophyletic clade with strong support by the minimum-evolution, maximum-likelihood, and Bayesian methods, while the clades were more distantly related in the analysis by Blair et al. (42) (Fig. 2a and b). The addition of four mitochondrial markers (*cox2*, *nad9*, *rps10*, and *secY*) in a later 11-locus analysis by Martin et al. (47), while topologically supporting the data from Blair et al. (42), displayed poor resolution for many interclade relationships (particularly for more extensively derived clades such as clades 1 to 5) within *Phytophthora* by the maximum-likelihood, maximum-parsimony, and Bayesian methods (Fig. 2c). A coalescent approach using a similar data set by the same authors showed improved Bayesian support among some *Phytophthora* clades (e.g., clades 1 to 5) but weaker support for other clades and a conflicting topology from the 11-locus analysis (47) (Fig. 2d).

Placement of other taxa within the *Peronosporales* order, namely, the “downy mildews,” and the phylogeny of *Pythium* and the *Pythiales* order have also been difficult to resolve. The inclusion of two downy mildews species (*Hyaloperonospora arabidopsidis* and *Pseudoperonospora cubensis*) in an analysis conducted by Runge et al. placed the two species within *Phytophthora* clade 4 and sister to clade 1 species such as *Phytophthora infestans*, implying the existence of a paraphyletic *Phytophthora* genus (46) (Fig. 2b). However, a subsequent tree reconciliation analysis, inferred using a class phylogeny of 189 oomycete clusters of orthologous groups (COGs), placed *H. arabidopsidis* as sister to members of the *Phytophthora* genus (48). Another downy mildew species, *Plasmopara halstedii*, was placed sister to *Phytophthora* clade 1 in similar phylogenetic analyses (36, 49). *Phytopythium*, a morphological intermediate between *Phytophthora* and *Pythium*, was reclassified from *Pythium* clade K to its own genus within the *Peronosporales* order based on a recent multigene phylogenetic analysis which placed the genus sister to *Phytophthora* (50). *Pythium* itself is divided into 10 clades, labeled A to J, which were initially circumscribed with its data and consistent with mitochondrial data (51). The main morphological difference between clades within *Pythium* is the development of the filamentous sporangium in species within clades A to C from the ancestral globose sporangium observed in the basal clades and *Phytopythium* (51, 52), with an intermediate contiguous sporangium developing in species within clade D (51) and an elongated sporagium in species within clade H (53). Otherwise, as in *Phytophthora*, phylogenetic clades generally do not correlate with distinct morphological characters in *Pythium* (51). A number of phylogenetic analyses suggest that *Pythium* is polyphyletic (36, 49, 52–55), and there has been recent suggestion that it be amended entirely into at least five new genera (53, 56).

Many of the aforementioned phylogenetic analyses of the oomycetes are based upon a small number of highly conserved nuclear and/or mitochondrial markers, either through consensus analysis or concatenated analysis. The selection of such markers, while usually robust, may unintentionally ignore other types of potential phylogenetic markers that might resolve conflicting analyses, such as lineages which include gene duplication events (20). One solution to the possible limitations of single-gene or small-scale gene phylogenies is to assemble a consensus phylogeny for a given set of taxa using many sources of single-gene phylogenies through supertree analysis, which enables the inclusion of phylogenies with missing or duplicated taxa (57). Matrix representation using parsimony (MRP), in which character matrices are generated for each source phylogeny and merged into a single binary character matrix for maximum-parsimony alignment (58, 59), is one of the most commonly used supertree methods and has seen successful application in a number of eukaryotic phylogenomic studies (60–62). Other methods have been developed for inferring species phylogeny from paralogous gene phylogenies, the most successful of which has been gene tree parsimony (GTP) (63). GTP attempts to find the most parsimonious species tree from a set of source phylogenies with the lowest number of events required to explain incongruences (i.e., gene duplication events) between the source phylogenies and has seen application in large-scale phylogenetic analysis (64). Another method of large-scale phylogenetic analysis is the supermatrix approach of concatenating multiple character data sets for simultaneous analysis (65).

Since the publication of the genome sequences of *Phytophthora sojae* and *Phytophthora ramorum* in 2006 (66), the quantity of oomycete genomic data has steadily increased; currently, 37 oomycete species now have publicly available genomic data at the assembly level or higher (Table 1). With this in mind, we have conducted the first whole-genome phylogenetic analysis for the oomycetes as a class, using a variety of supertree and supermatrix approaches which have previously been used in fungal whole-genome phylogenetic analysis (60). In our analysis, we utilized protein data from 37 complete oomycete genomes and 6 complete SAR genomes (as outgroups). This represents all extant genomic data from the four “crown” oomycete orders and covers 8 of the 10 phylogenetic clades within *Phytophthora* and 7 of the 10 phylogenetic clades within *Pythium* (Table 1). Our whole-genome phylogenetic analysis of the oomycetes supports the four oomycete orders and the placement of *Phytopythium* within the *Peronosporales* and individual clades within *Phytophthora* and *Pythium*. The resolution of the *Peronosporales* as an order varied under different methods, probably due to missing data from clades 4 and 9 within *Phytophthora*. However, the overall order phylogenies are relatively congruent among our different species phylogenies. This analysis will provide a useful backbone to future genome phylogenies of the oomycetes utilizing more taxonomically extensive data sets.

**TABLE 1:** Taxonomic and genomic information for the 43 oomycete and SAR species in this analysis^a^

Species name	Clade	Order	Class	Reference	Gene
*Albugo candida*	NA	*Albuginales*	*Oomycota*	Links et al. 2011 (73)	13310
*Albugo labiachii*	NA	*Albuginales*	*Oomycota*	Kemen et al. 2011 (74)	13804
					
*Hyaloperonospora arabidopsidis*	NA	*Peronosporales*	*Oomycota*	Baxter et al. 2010 (71)	14321
*Phytophthora agathidicida*	Clade 5	*Peronosporales*	*Oomycota*	Studholme et al. 2016 (70)	14110*
*Phytophthora capsici*	Clade 2	*Peronosporales*	*Oomycota*	Lamour et al. 2012 (72)	19805
*Phytophthora cinnamomi*	Clade 7	*Peronosporales*	*Oomycota*	Studholme et al. 2016 (70)	12942*
*Phytophthora cryptogea*	Clade 8	*Peronosporales*	*Oomycota*	Feau et al. 2016 (75)	11876*
*Phytophthora fragariae*	Clade 7	*Peronosporales*	*Oomycota*	Gao et al. 2015 (76)	13361*
*Phytophthora infestans*	Clade 1	*Peronosporales*	*Oomycota*	Haas et al. 2009 (69)	17797
*Phytophthora kernoviae*	Clade 10	*Peronosporales*	*Oomycota*	Sambles et al. 2015 (77)	10650
*Phytophthora lateralis*	Clade 8	*Peronosporales*	*Oomycota*	Quinn et al. 2013 (78)	11635
*Phytophthora multivora*	Clade 2	*Peronosporales*	*Oomycota*	Studholme et al. 2016 (70)	15006*
*Phytophthora nicotianae*	Clade 1	*Peronosporales*	*Oomycota*	Liu et al. 2016 (79)	10521
*Phytophthora parasitica*	Clade 1	*Peronosporales*	*Oomycota*	Broad Institute (INRA-310 v. 3)	27942
*Phytophthora pinifolia*	Clade 6	*Peronosporales*	*Oomycota*	Feau et al. 2016 (75)	19533*
*Phytophthora pluvialis*	Clade 3	*Peronosporales*	*Oomycota*	Studholme et al. 2016 (70)	18426*
*Phytophthora pisi*	Clade 7	*Peronosporales*	*Oomycota*	PRJEB6298	15495*
*Phytophthora ramorum*	Clade 8	*Peronosporales*	*Oomycota*	Tyler et al. 2006 (66)	15743
*Phytophthora rubi*	Clade 7	*Peronosporales*	*Oomycota*	PRJNA244739	15462*
*Phytophthora sojae*	Clade 7	*Peronosporales*	*Oomycota*	Tyler et al. 2006 (66)	26584
*Phytophthora* taxon Totara	Clade 3	*Peronosporales*	*Oomycota*	Studholme et al. 2016 (70)	16691*
*Plasmopara halstedii*	NA	*Peronosporales*	*Oomycota*	Sharma et al. 2015 (80)	15469
*Plasmopara viticola*	NA	*Peronosporales*	*Oomycota*	PRJNA329579	12048*
*Phytopythium vexans*	NA	*Peronosporales*	*Oomycota*	Adhikari et al. 2013 (67)	11958
					
*Pilasporangium apinafurcum*	NA	*Pythiales*	*Oomycota*	PRJDB3797	13184*
*Pythium aphanidermatum*	Clade A	*Pythiales*	*Oomycota*	Adhikari et al. 2013 (67)	12312
*Pythium arrhenomanes*	Clade B	*Pythiales*	*Oomycota*	Adhikari et al. 2013 (67)	13805
*Pythium insidiosum*	Clade C	*Pythiales*	*Oomycota*	Rujirawat et al. 2015 (81)	19290*
*Pythium irregulare*	Clade F	*Pythiales*	*Oomycota*	Adhikari et al. 2013 (67)	13805
*Pythium iwayami*	Clade G	*Pythiales*	*Oomycota*	Adhikari et al. 2013 (67)	14875
*Pythium oligandrum*	Clade D	*Pythiales*	*Oomycota*	Berger et al. 2016 (82)	14292*
*Pythium ultimum* var. *sporangiiferum*	Clade I	*Pythiales*	*Oomycota*	Adhikari et al. 2013 (67)	14096
*Pythium ultimum* var. *ultimum*	Clade I	*Pythiales*	*Oomycota*	Lévesque et al. 2010 (68)	15323
					
*Aphanomyces astaci*	NA	*Saprolegniales*	*Oomycota*	Broad Institute (APO3 v.2)	26259
*Aphanomyces invadans*	NA	*Saprolegniales*	*Oomycota*	Broad Institute (9901 v.2)	20816
*Saprolegnia diclina*	NA	*Saprolegniales*	*Oomycota*	PRJNA168273	18229
*Saprolegnia parasitica*	NA	*Saprolegniales*	*Oomycota*	Jiang et al. 2013 (83)	20121
					
*Aureococcus anophagefferns*	NA	*Pelagomonadales*	*Pelagophyceae*	Gobler et al. 2011 (84)	11501
*Ectocarpus siliculosus*	NA	*Ectocarpales*	*Phaeophyceae*	Cock et al. 2010 (87)	16269
*Phaeodactylum tricornutum*	NA	*Naviculales*	*Bacillariophyceae*	Bowler et al. 2008 (85)	10402
*Thalassiosira psuedonana*	NA	*Thalassiosirales*	*Coscinodiscophyceae*	Armbrust et al. 2004 (86)	11776
					
*Paramecium tetraurelia*	NA	*Peniculida*	*Oligohymenophorea*	Aury et al. 2006 (88)	39580
*Bigelowiella natans*	NA	*Chlorarachniophyceae*	*Cercozoa*	Curtis et al. 2012 (89)	21708

a Protein counts generated in this study from assembly data are highlighted with an asterisk (*). References are to the genome publications where possible and otherwise to the NCBI BioProject identifier or the Broad Institute strain identifier and assembly version. NA, not applicable.

## RESULTS AND DISCUSSION

### Identification of orthologous and paralogous oomycete and SAR gene families.

For our supertree analyses, we constructed a data set containing 43 complete genomes, consisting of 37 from oomycete species and 6 outgroups from other species within the SAR supergroup (Materials and Methods; Table 1). Of these 37 oomycete genomes, 26 were from either *Phytophthora* species or *Pythium* species representing the majority of clades within both genera, and the remainder were sampled from all four of the “crown” orders (66–89). We downloaded proteomes for 23 oomycete species which were available from public databases, and we generated corresponding proteomes for the remaining 14 species from publicly available assembly data using bespoke oomycete reference templates with AUGUSTUS and GeneMark-ES (90, 91) (Table S1). In total, our final data set contained 702,132 protein sequences from 37 complete oomycete genomes and 6 complete SAR genomes (Table 1).

10.1128/mSphere.00095-17.4 TABLE S1  Taxonomy, assembly statistics, and protein predictions for 14 oomycete species. AUGUSTUS *ab initio* protein predictions were carried out using parameters generated from genomic, EST, and protein data from template species. Protein prediction for *Pi. apinafurcum* was carried out using GeneMark-ES and AUGUSTUS. Protein sets used in whole-genome phylogenetic analyses in this study are highlighted in bold. Download TABLE S1, DOCX file, 0.02 MB. Copyright © 2017 McCarthy and Fitzpatrick. 2017 McCarthy and Fitzpatrick This content is distributed under the terms of the Creative Commons Attribution 4.0 International license.

The initial step in determining the phylogeny of the 43 oomycete and SAR genomes in our data set through supertree methods was to identify groups of closely related orthologs or paralogs within our data set, which we termed gene families, and to use these groups to generate gene phylogenies to use as source data for our methods. To identify families of orthologous and paralogous genes in our data set, we set the following criteria:

(1) A single-copy gene family must contain no more than one orthologous gene per species and must be present in four or more species.
(2) A multicopy gene family must contain at least four unique species, and two or more paralogs must be present in at least one of the species.

Using OrthoMCL (92), with an inflation value of 1.5 and a strict BLASTp cutoff value of 10^−20^ (93) and bespoke Python scripting, we identified over 56,000 homologous oomycete and SAR gene families in our data set. Of these, 2,853 families matched our criterion for single-copy families and 11,158 families matched our criterion for multicopy families. By aligning each of these gene families in MUSCLE (94) and sampling for highly conserved regions using Gblocks (95), both using the default parameters, and then carrying out permutation-tail possibility (PTP) tests for every remaining sampled alignment using PAUP* (96, 97), we were able to remove 576 single-copy gene families and 5,103 multicopy gene families with poor phylogenetic signal from our data. All remaining gene families had their evolutionary model estimated using ProtTest (98) (Table S2), and maximum-likelihood gene phylogenies were generated using PhyML with 100 bootstrap replicates (99). We generated phylogenetic reconstructions for 2,280 orthologous gene families (containing 35,622 genes) and 6,055 paralogous gene families (containing 174,282 genes). In total, from our 43-genome data set, we identified 8,335 individual gene phylogenies, containing 209,904 oomycete and SAR genes.

10.1128/mSphere.00095-17.5 TABLE S2  Selection of evolutionary models (not including additional parameters) for single-copy and multicopy gene phylogenies by ProtTest. Download TABLE S2, DOCX file, 0.01 MB. Copyright © 2017 McCarthy and Fitzpatrick. 2017 McCarthy and Fitzpatrick This content is distributed under the terms of the Creative Commons Attribution 4.0 International license.

### Supetree phylogenies fully resolve oomycete class and order phylogenies.

**FIG 3:**
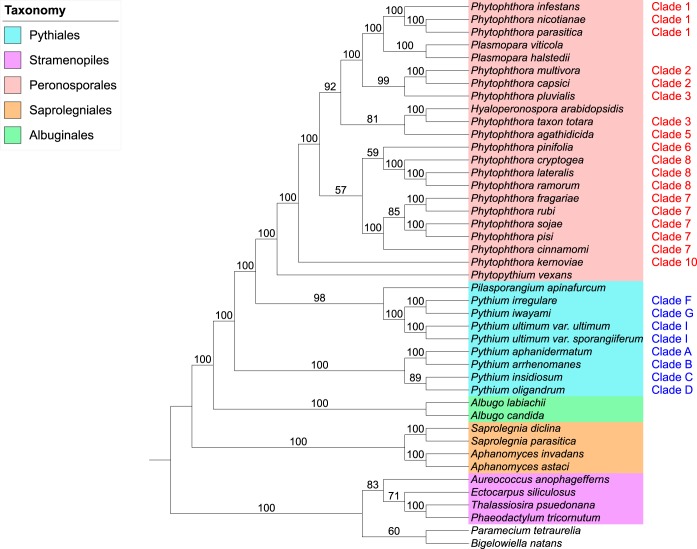
Matrix representation with parsimony (MRP) supertree of 37 oomycete species and 6 SAR species (2,280 source phylogenies). The supertree was generated in CLANN. The phylogeny is rooted at the SAR branch. *Phytophthora* clades as designated by Blair et al. (42) and *Pythium* clades as designated by de Cock et al. (50) are indicated in red and blue, respectively. No color, *P. tetraurelia* (*Alveolata*) and *B. natans* (*Rhizaria*).

All 2,280 orthologous single-copy gene phylogenies (35,622 genes in total) were used as input for CLANN (100), which implements a matrix representation using parsimony (MRP) method to determine consensus phylogeny for many source phylogenies with overlapping taxa or missing taxa. An MRP supertree phylogeny was generated in CLANN using a heuristic search with 100 bootstrap replicates. The supertree was visualized and annotated within the Interactive Tree of Life (iTOL) website (101) and rooted at the branch containing the SAR outgroups, *Paramecium tetraurelia* (*Alveolata*), *Bigelowiella natans* (*Rhizaria*), and four stramenopiles species (Fig. 3).

**FIG 4:**
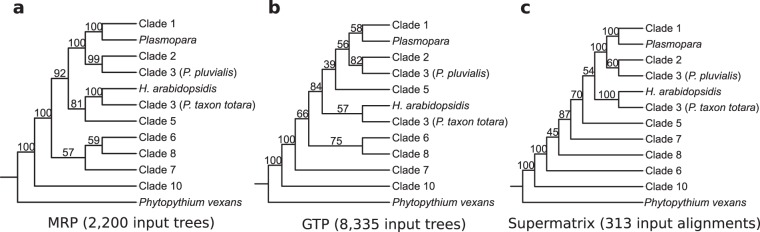
Congruence of the *Peronosporales* order data between our supertree and supermatrix methods. (a) MRP analysis. (b) GTP analysis. (c) Concatenated supermatrix analysis. For full phylogenies, refer to Fig. 3, 5, and 6, respectively.

MRP supertree analysis of 2,280 orthologous single-copy oomycete gene phylogenies supported the four “crown” oomycete orders (*Saprolegniales*, *Albuginales*, *Pythiales*, and *Peronosporales*), with maximum bootstrap support (BP) (Fig. 3). The MRP supertree reflects the consensus phylogeny of the oomycetes (31–33) (Fig. 1). The *Saprolegniales* species represent the most basal “crown” order, and the *Albuginales* is a sister order to the *Pythiales* and *Peronosporales*. Within the *Pythiales* themselves, a highly supported split among *Pythium* clades A to D (100% BP) and clades F to I (100% BP) was observed, matching similar splits seen in small-scale analyses (51, 52) (Fig. 3). *Pilasporangium apinafurcum*, a *Pythiales* species, is placed sister to *Pythium* clades F to I (98% BP). *Phytopythium vexans* is placed at the base of the *Peronosporales* order (Fig. 3), supporting the recent reclassification of the *Phytopythium* genus from the *Pythiales* (50). Many individual *Phytophthora* clades within the *Peronosporales* are well supported. In addition, the “downy mildews” species in our data set (*Hyaloperonospora arabidopsidis* and two *Plasmopara* species) place as derived taxa within the *Peronosporales* order rather than as basal to *Phytophthora* (Fig. 3). The overall phylogeny of the *Peronosporales* in our MRP supertree is summarized in Fig. 4a and discussed in greater detail later in the text. As an additional analysis, a consensus supernetwork of the phylogenetic splits within the 2,280 single-copy gene phylogenies was generated in SplitsTree (102) (see Fig. S1 in the supplemental material). The network further highlights support for the four “crown” oomycete orders and the division of the *Pythiales* order as in the supertree phylogeny; it also recapitulates many of individual *Phytophthora* clades and intraorder relationships within the *Peronosporales* (Fig. 3 and 4a; Fig. S1).

10.1128/mSphere.00095-17.1 FIG S1  Consensus network of phylogenetic splits within 2,280 single-copy oomycete and SAR phylogenies. The network was generated in SplitsTree. Clades are colored by taxonomic order as follows: red, *Peronosporales*; blue, *Pythiales*; green, *Albuginales*; orange, *Saprolegniales*; no color, *P. tetraurelia* (*Alveolata*) and *B. natans* (*Rhizara*). Download FIG S1, PDF file, 0.1 MB. Copyright © 2017 McCarthy and Fitzpatrick. 2017 McCarthy and Fitzpatrick This content is distributed under the terms of the Creative Commons Attribution 4.0 International license.

**FIG 5:**
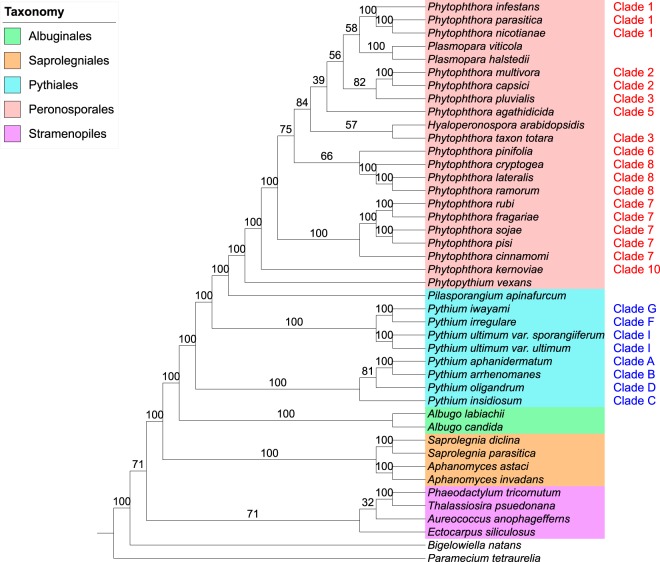
Gene tree parsimony (GTP) supertree of 37 oomycete species and 6 SAR species (8,335 source phylogenies). The supertree was generated in DupTree. The phylogeny is rooted at the SAR branch. *Phytophthora* clades as designated by Blair et al. (42) and *Pythium* clades as designated by de Cock et al. (50) are indicated in red and blue, respectively. No color, *P. tetraurelia* (*Alveolata*) and *B. natans* (*Rhizaria*).

Both the 2,280 single-copy phylogenies and the 6,055 multicopy phylogenies (209,904 genes in total) were used as input for DupTree (103), which uses a gene tree parsimony (GTP) method to determine consensus phylogeny for many source phylogenies that may include gene duplication events. The source data were bootstrapped with 100 replicates, and the resultant consensus GTP supertree was rooted at the branch containing *Paramecium tetraurelia*, *Bigelowiella natans*, and the other stramenopiles species (Fig. 5). As in the single-gene MRP supertree, all four individual crown oomycete orders and the oomycete class phylogeny are highly supported. The *Pythiales* order is once again split into highly supported sister branches containing clades A to D (100% BP) and clades F to I (100% BP) (Fig. 5). The *Peronosporales* order is highly supported again (100% BP), as is the placement of *Phytopythium vexans* at the base of this order (Fig. 5). As with the single-gene MRP supertree, the downy mildews (*P. viticola* and *P. halstedii*) are found as sister taxa to clade 1 *Phytophthora* species. However, it is worth pointing out that phylogenetic support for this grouping is weaker in the GTP supertree (58% BP) (Fig. 4b and 5) than in the MRP supertree, where support is very strong (100% BP) (Fig. 3). Overall, the phylogeny of the *Peronosporales* order in our GTP supertree displays weaker bootstrap support at some branches than in the single-gene MRP supertree. However, with the exception of the placement of clade 5, the overall taxonomic congruence between the two supertree approaches for the *Peronosporales* is high (Fig. 3, 4a and b, and 5).

### The supermatrix approach based on ubiquitous *Peronosporales* gene phylogenies supports supertree phylogenies.

As a complement to our supertree method phylogenies, we undertook a supermatrix approach to infer the oomycete species phylogeny using oomycete orthologs of known proteins corresponding to clusters of orthologous groups (COG) as phylogenetic markers (104). To identify oomycete COGs, we performed a reciprocal BLASTp analysis of all 458 *Saccharomyces cerevisiae* COGs against the 37 oomycete proteomes in our full data set (590,896 protein sequences in total) with an E value of 10^−10^. Overall, 443 oomycete gene families that were reciprocal top hits to *S. cerevisiae* COGs were retrieved. Of the 443 COG families, 144 families contained an ortholog from all 37 oomycete species and were retained for analysis. A superalignment of 16,934 characters was generated by concatenating the 131 aligned families which retained alignment data after Gblocks sampling with FASconCAT (105). The maximum-likelihood phylogeny of this superalignment was reconstructed in PhyML with 100 bootstrap replicates and an LG+I+G+F amino acid substitution model as selected by ProtTest, and the resultant consensus phylogeny was rooted at the *Saprolegniales* branch (Fig. S2). This initial supermatrix phylogeny supported the four “crown” orders similarly to our supertree phylogenies; however, poor resolution and inconsistent phylogeny were observed within the *Peronosporales*, particularly the placement of species from *Phytophthora* clades 7 and 8; for example, clade 7 species are not monophyletic (Fig. S2). To attempt to tease apart the data corresponding to the poor resolution of the *Peronosporales* in our maximum-likelihood phylogeny, a neighbor-joining network was generated for the COG superalignment in SplitsTree to visualize the bifurcations within the superalignment (Fig. S3). As can be seen in the network, a significant amount of phylogenetic conflict is obvious and is represented as alternative splits among *Peronosporales* clades, a phenomenon that is consistent with poor bootstrap support and inconsistent topology (relative to supertrees) throughout the *Peronosporales* in this class-level supermatrix phylogeny (Fig. S2 and S3).

10.1128/mSphere.00095-17.2 FIG S2  Maximum-likelihood (ML) supermatrix phylogeny of 37 oomycete species (144 families of *Saccharomyces cerevisiae* COG homologs, 16,934 characters). Supermatrix phylogeny data were generated in PhyML with an LG+I+G+F amino acid substitution model. The phylogeny is rooted at the *Saprolegniales* branch. *Phytophthora* clades as designated by Blair et al. (42) and *Pythium* clades as designated by de Cock et al. (50) are indicated in red and blue, respectively. No color, *P. tetraurelia* (*Alveolata*) and *B. natans* (*Rhizaria*). Download FIG S2, PDF file, 0.1 MB. Copyright © 2017 McCarthy and Fitzpatrick. 2017 McCarthy and Fitzpatrick This content is distributed under the terms of the Creative Commons Attribution 4.0 International license.

10.1128/mSphere.00095-17.3 FIG S3  Neighbor-joining network of phylogenetic splits within oomycete COG supermatrix of 37 oomycete species. The network was generated in SplitsTree. Clades are colored by taxonomic order as follows: red, *Peronosporales*; blue, *Pythiales*; green, *Albuginales*; orange, *Saprolegniales*. Download FIG S3, PDF file, 0.02 MB. Copyright © 2017 McCarthy and Fitzpatrick. 2017 McCarthy and Fitzpatrick This content is distributed under the terms of the Creative Commons Attribution 4.0 International license.

**FIG 6:**
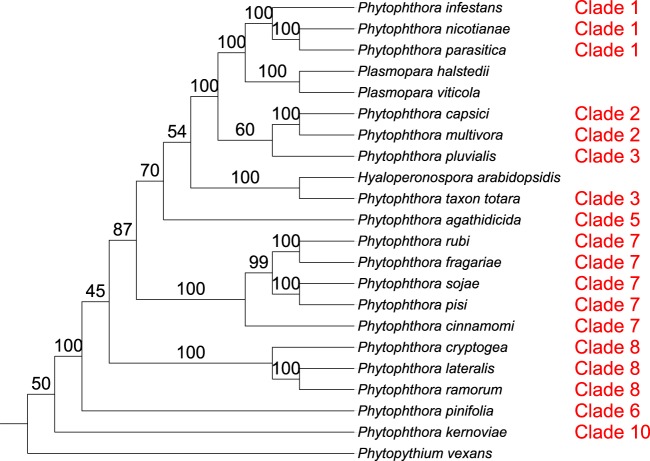
Maximum-likelihood (ML) supermatrix phylogeny of 22 *Peronosporales* species (313 ubiquitous *Pernosporales* gene families, 47,635 characters). The supermatrix phylogeny was generated in PhyML with a JTT+I+G+F amino acid substitution model. The cladogram is rooted at *Phytopythium vexans*. *Phytophthora* clades as designated by Blair et al. (2008) are shown in red.

To extend our COG supermatrix phylogeny, we took the approach of generating a supermatrix from ubiquitous gene families within the 22 *Peronosporales* species in our data set. Using this approach, we hoped to extend the amount of available alignment data for species solely within *Peronosporales* to improve resolution of the order. We defined a ubiquitous *Peronosporales* gene family as containing exactly one ortholog from all 22 *Peronosporales* species in our data set. Using OrthoMCL, with a strict BLASTp E value of 10^−20^ and an inflation value of 1.5, we identified over 20,000 orthologous gene families in the 22 *Peronosporales* proteomes in our data set. From these families, we identified 352 ubiquitous gene families within *Peronosporales* using bespoke Python scripting; each family was then aligned in MUSCLE and sampled in Gblocks. After removing families which did not retain alignment data after Gblocks, we concatenated the remaining 313 gene families into a superalignment that was 47,365 amino acids in length. The maximum-likelihood phylogeny for this superalignment was generated with 100 bootstrap replicates and a JTT+I+G+F evolutionary model. The resultant consensus phylogeny was rooted at *Phytopythium vexans* (Fig. 6). While resolution of relationships among clades is still weak at some branches, the higher support seen on many other branches and the overall topology of the ubiquitous supermatrix phylogeny represent substantial improvements over the COG supermatrix. *Phytophthora* clades 1, 2, 7, and 8 are now all monophyletic, with 100% bootstrap support each. The genus is split between the basal lineages (*Phytopythium* and *Phytophthora* clades 6 to 10) and the more extensively derived *Phytophthora* clades (clades 1 to 5) and the downy mildews, which form a monophyletic group (70% BP) (Fig. 4c and 6), an inference that is also observed in our supertree species phylogenies and with the highest degree of congruence to the single-gene MRP supertree (Fig. 4a and b).

### Resolution of the *Peronosporales* order in phylogenomic analysis.

All three of our whole-genome species phylogenies strongly support the *Peronosporales* order (Fig. 4) and display a high degree of congruence with one another. Each phylogeny also supports the recent reclassification of *Phytopythium* from the *Pythiales* to the *Peronosporales* as a basal taxon (50). All three phylogenies also show varying but strong bootstrap support (70 to 92% BP) for the divergence of *Phytophthora* clades 1 to 5 and the downy mildews (*Plasmopara* spp., *H. arabidopsidis*) from the remaining *Phytophthora* clades and *Phytopythium* at a single point (Fig. 4c). The relationships among these taxa across our phylogenies can be summarized as follows:

(1) The downy mildews species *Hyaloperonospora arabidopsidis* and *Phytophthora* taxon Totara (*Phytophthora* clade 3) are sister taxa, with maximum support in both MRP and supermatrix analysis (Fig. 4a and c). Therefore, *Phytophthora* clade 3 is not monophyletic in any of our species phylogenies (Fig. 4). *Phytophthora* taxon Totara has provisionally been assigned to clade 3 based on sequence similarity. Our species phylogenies suggest that it is not actually a clade 3 species.
(2) A close relationship between *Phytophthora* clades 1 and 2, the clade 3 species *Phytophthora pluvialis*, and the downy mildew species *Plasmopara viticola* and *Plasmopara halstedii* is observed in each phylogeny, with maximum support in both MRP and supermatrix analysis (Fig. 4a and c).

The placement of the clade 5 species *Phytophthora agathidcida* varies in each phylogeny, but it appears that the species is most closely related to *Phytophthora* taxon Totara and *H. arabidopsidis* within the *Peronosporales*, as is most apparent in the single-gene MRP supertree (81% BP) (Fig. 3 and 4a). As for the more basal clades, both the MRP and GTP phylogenies show support for the idea of clade 6 species *Phythophthora pinifolia* being sister to *Phytophthora* clade 8, with highest bootstrap support of 59% and 75%, respectively (Fig. 4a and b).

In our analysis, we set out to resolve relationships within the oomycetes where conflicts have arisen in different analyses, particularly in the *Peronosporales* order (Fig. 2). With respect to the divergence of *Phytophthora* clades 1 to 5 and the downy mildews from the remaining basal taxa in the *Peronosporales* (i.e., *Phytophthora* clades 6 to 10 and *Phytopythium*), our results are congruent with the small-scale analyses performed by Blair et al. and Martin et al. (42, 47) (Fig. 2a, c, and d), with closest topological similarity to the latter authors’ 6-locus coalescence method phylogeny (Fig. 2d), despite a lack of data from *H. arabidopsidis* and *Plasmopara* species in both analyses and the inclusion of *H. arabidopsidis* data in the analysis carried out by Runge et al. (46) (Fig. 2b). Our own analysis lacks data from any species in *Phytophthora* clade 4, which is still unsampled in terms of genome sequencing. In the analysis by Runge et al., *H. arabidopsidis* branches within paraphyletic *Phytophthora* clade 4; were there a representative species from clade 4 available, a greater degree of resolution for the relationships among *Phytophthora* clades 3 to 5 and *Hyaloperonospora* might be observed. However, it is not clear whether the placement of *H. arabidopsidis* relative to *Phytophthora* clade 1 would then recapitulate that described by Runge et al. (46). Similarly, with regard to the basal taxa, our result are relatively congruent with the linearized relationships seen in previous analyses (Fig. 2), although the close relationship of clade 6 species *Phytophthora pinifolia* to *Phytophthora* clade 7 seen in our two supertree methods is not reflected in any of the multilocus phylogenies (Fig. 4a and b). The resolution of the relationships among *Phytophthora* clades 6, 7, and 8 varies both in support and sister group relationships among our analyses (Fig. 4); however, similar variation can be observed between the highlighted multilocus phylogenies (42, 46, 47) (Fig. 2). The lack of available genomic data from *Phytophthora* clade 9 also prevents any conclusions regards its placement in a whole-genome phylogeny; however, we would expect that it would branch as a sister to clade 10 species such as *Phytophthora kernoviae*, as the relationship between clades 9 and 10 has been highly supported in multilocus analyses (42, 46, 47).

### The use of supertree and phylogenomic methods in oomycete systematics.

Our analysis is the first large-scale genome phylogeny of the oomycetes as a class, using all extant genomic data from 37 oomycete species. Our analysis has recapitulated the four crown orders of the oomycetes and many relationships within the two largest-sampled orders, the *Pythiales* and the *Peronosporales*. During our analysis, we were conscious of potential characteristics of oomycete genomes that could obfuscate phylogenomic analysis. The role of HGT and its impact on the quality of our analyses were considered; it has been shown that supertree and supermatrix analyses are thought to be susceptible to misleading signal in data sets where a large degree of HGT has occurred, particularly in MRP analysis (106). While HGT from other microbial eukaryotes, fungi, and prokaryotes has been identified within oomycete genomes, the majority of these events are thought to be ancestral or to have not occurred in proportions large enough to impact our results (4, 5, 107). Other factors, such as fast-evolving regions of genomes or ancestral gene loss or duplication events within the oomycetes, are not likely to have affected our analysis, given our genome-wide scale of data acquisition and our strict filtering of gene families with poor phylogenetic signal (10, 48, 96). Intraspecific hybridization within the *Phytophthora* genus has been increasingly reported in the literature and usually occurs in nature among *Phytophthora* species within the same phylogenetic clade (108). A number of hybrid species or hybridization events have been described in *Phytophthora* clades 6 to 8 (108–110); however, none of these species are present in our data set. Also, where hybridization has occurred, it has been between closely related species and, in the case of *Phytophthora* species, those from the same phylogenetic clade. Taking this into consideration, hybridization should affect intraclade relationships to a greater degree than interclade relationships.

Compared with fungi, particularly in light of the ongoing 1,000 fungal genomes project (http://1000.fungalgenomes.org), there is a relative dearth of genomic data for both the earliest diverging lineages and the “crown” taxa within the oomycetes. With the greater sampling of genomic sequencing of the oomycetes likely to occur in the future, it is our view that subsequent genome phylogenies of the oomycetes will match the success of other eukaryotic genome phylogenies at resolving individual problematic clade and species relationships (60, 62). We suspect that, with a broader sampling of all *Phytophthora* clades and downy mildew species, we would see better resolution of the *Peronosporales* within any subsequent oomycete genome phylogenies. Similar approaches with other oomycete taxa, such as *Pythium*, may disentangle some of the phylogenetic conflicts seen in recent analyses (49, 53). Similarly, sequencing of more *Saprolegniales* species or basal oomycete species and their inclusion in similar analyses will potentially help uncover further aspects of oomycete evolution, including the evolution of phytopathogenicity. Such analyses, for which ours is a first step, would also provide the benefit of establishing a robust phylogeny for a eukaryotic group with such devastating ecological impact and would hopefully encourage further genomics and phylogenomics research into the oomycetes.

### Conclusions.

Using 37 oomycete genomes and 6 SAR genomes, we have carried out the first whole-genome phylogenetic analysis of the oomycetes as a class. The different methods that we used in our analysis support the four “crown” oomycete orders and support many individual phylogenetic clades within genera. Our analysis also generally supports the placement of *Phytopythium* within the *Peronosporales*, the placement of the downy mildews within the *Phytophthora* genus, and the topology of clades within the *Pythiales* order. However, resolution of the *Peronosporales* as an order remains weak at some branches, possibly due to a lack of genomic data for some phylogenetic clades within *Phytophthora*. As the amount of genomic data available for the oomycetes increases, future genome phylogenies of the class should resolve these branches, as well as those within currently unsampled basal lineages or undersampled taxa such as *Saprolegnia*. Our analysis represents an important backbone for oomycete phylogenetics upon which future analyses can be based.

## MATERIALS AND METHODS

### Data set assembly.

The predicted proteomes for 29 SAR species (23 oomycete species, 4 other stramenopile species, the alveolate species *Paramecium tetraurelia*, and the rhizarian species *Bigelowiella natans*) were obtained from public databases (Table 1). Predicted proteomes for a further 14 oomycete species (10 *Phythophthora* species, 2 *Pythium* species, *Plasmopara viticola*, and *Pilasporangium apinafurcum*) were generated from publically available assembly data using AUGUSTUS (90). Templates for *ab initio* protein prediction with AUGUSTUS were generated from assembly and expressed sequence tag (EST) data from a number of reference oomycete species (*Phytophthora sojae*, *Phytophthora capsici*, *Pythium ultimum* var. *ultimum*, and *Plasmopara halstedii*) (Table S1). *Ph. capsici* was used as a reference for *Phytophthora* species from clades 1 to 5, while *Ph. sojae* was used as a reference for *Phytophthora* species from clades 6 to 10. *Py. ultimum* var. *ultimum* was used as a reference for two *Pythium* species and *Pi. apinafurcum*. *P. halstedii* was used as a reference for *P. viticola*. GeneMark-ES (91) was used in conjuction with AUGUSTUS for protein prediction for *Pi. apinafurcum*. The taxonomy, assembly, and prediction statistics for each of the 14 assemblies included in this study are summarized in Table S1. Our final data set contained 702,132 protein sequences from 37 oomycete genomes and 6 SAR genomes (66–89) (Table 1; Table S1).

### Identification and reconstruction of gene phylogenies in oomycete and SAR genomes.

All 702,132 protein sequences in our data set were filtered and clustered into 56,638 orthologous gene families using OrthoMCL (92), with a BLASTp E value cutoff of 10^−20^ (93) and an inflation value of 1.5. Using bespoke Python scripting, we identified and retrieved two types of gene family containing 200 sequences or fewer from the 56,638 families within our data set as follows:

(1) A total of 2,853 single-copy gene families (single-copy orthologs present in ≥4 species.
(2) A total of 11,158 multicopy gene families (≥1 paralog[s] present in ≥4 species).

Each of these gene families was retrieved and aligned in MUSCLE (94), and highly conserved regions of these alignments were sampled using Gblocks (95) with the default parameters. A total of 266 single-copy gene families and a total of 4,928 multicopy gene families did not retain alignment data after Gblocks sampling and were discarded. Permutation-tail probability (PTP) tests (96) were carried out for every remaining sampled gene family in PAUP* (97), using 100 replicates, to determine whether a given sampled gene family had phylogenetic signal. Those sampled gene families whose PTP test result had a *P* value of ≤0.05 were considered to have signal and were retained. A total of 2,280 single-copy sampled gene families (containing 35,622 genes in total) and a total of 6,055 multicopy sampled gene families (containing 174,282 genes in total) ultimately satisfied our filtering process. Best-fit amino acid replacement models were selected for every remaining sampled gene family using ProtTest (Table S2), and maximum-likelihood phylogenetic reconstruction was carried out using PhyML with 100 bootstrap replicates.

### Supertree analyses of single-copy and paralogous gene phylogenies.

Maximum-parsimony supertree analysis of 2,280 single-copy gene phylogenies (containing 35,622 genes in total) was carried out using CLANN, by performing a subtree prune and regraft (SPR) heuristic search with 100 bootstrap replicates (100). This phylogeny was visualized and annotated as a cladogram using the Interactive Tree of Life (iTOL) website (101) (Fig. 3). As an additional analysis, a consensus supernetwork of phylogenetic multifurcations within the 2,280 individual gene phylogenies was generated in SplitsTree (102) (see Fig. S1 in the supplemental material). Gene tree parsimony (GTP) supertree analyses of all 8,335 gene phylogenies (containing 209,904 genes in total) was carried out using DupTree (103) and a rooted SPR heuristic search of 100 bootstrapped replicates of each phylogeny. A consensus phylogeny was generated from all individual replicates and was visualized and annotated as a cladogram using iTOL (Fig. 5).

### Identification and supermatrix analysis of ubiquitous oomycete gene phylogenies.

A reciprocal BLASTp search was carried out with an E value cutoff of 10^−10^ among all 37 oomycetes proteomes in our data set (590,896 protein sequences in total) and 458 core orthologous genes (COGs) in *Saccharomyces cerevisiae* from the CEGMA data set (93, 104). A total of 443 oomycete gene families representing oomycete top hits to *S. cerevisiae* COGs were retrieved, among which 144 families contained an ortholog from all 37 oomycete species in our data set. Each of these 144 families was aligned in MUSCLE and was sampled for highly conserved regions using Gblocks with the default parameters. After 13 families which failed to retain alignment data after Gblocks sampling were removed, the remaining 131 sampled alignments (containing 4,847 genes in total) were concatenated into a superalignment of 16,934 aligned positions. This superalignment was bootstrapped 100 times using Seqboot, and maximum-likelihood phylogenetic trees were generated for each individual replicate using PhyML, with an LG+I+G+F amino acid substitution model as selected by ProtTest. A consensus tree was generated from these replicate trees using Consense, and the consensus tree was visualized and annotated as a cladogram using iTOL (Fig. S2). A neighbor-joining network of phylogenetic splits in the original superalignment was generated in SplitsTree (Fig. S3).

### Identification and supermatrix analysis of ubiquitous *Peronosporales* gene phylogenies.

A total of 347,375 protein sequences from the 22 *Peronosporales* proteomes in our data set were filtered and clustered into 22,803 orthologous gene families using OrthoMCL, with a BLASTp E value cutoff of 10^−20^ and an inflation value of 1.5. Using bespoke Python scripting, we identified 352 ubiquitous *Peronosporales* gene families, which we defined as any family which had exactly one representative ortholog from all 22 *Peronosporales* species in our data set. Each of these families was aligned in MUSCLE and sampled for highly conserved regions using Gblocks with the default parameters. After 39 gene families which did not retain alignment data after sampling were removed, the remaining 313 sampled alignments (containing 6,886 genes in total) were concatenated into a single superalignment of 47,365 aligned positions. This superalignment was bootstrapped 100 times using Seqboot, and maximum-likelihood phylogenetic trees were generated for each individual replicate using PhyML with a JTT+I+G+F amino acid substitution model, as selected by ProtTest. A consensus tree was generated from these replicate trees using Consense, and the consensus tree was visualized and annotated as a cladogram using iTOL (Fig. 6).
