# Supplementary material for: Phylogenomic Reconstruction of the Oomycete Phylogeny Derived from 37 Genomes
Source: mSphere. 2017 Apr 12;2(2):e00095-17. doi: 10.1128/mSphere.00095-17 (PMC5390094; doi:10.1128/mSphere.00095-17)
Supplement: TABLE S1 [file sph002172267st4.docx]

**Table S1**

| **Species** | **Clade** | **Subclade** | **Size (Mb)** | **Scaffolds** | **Contigs** | **GC (%)** | **Proteins predicted by AUGUSTUS** | |
| --- | --- | --- | --- | --- | --- | --- | --- | --- |
|  | | | | | | | **with *P. capsici*** | **with *P. sojae*** |
| *Phytophthora multivora* | 2 | - | 40 | 2,762 | 2,844 | 51.9 | **15006** | 8668 |
| *Phytophthora pluvialis* | 3 | - | 53 | 4,221 | 4,340 | 54.2 | **18426** | 12793 |
| *Phytophthora taxon totara* | 3 | - | 55 | 4,314 | 4,425 | 51.6 | **16691** | 10213 |
| *Phytophthora agathidicida* | 5 | - | 37 | 3,701 | 3,754 | 52.6 | **14110** | 8593 |
| *Phytophthora pinifolia* | 6 | - | 94 | 36,928 | 59,069 | 54.9 | 34341 | **19534** |
| *Phytophthora fragariae* | 7 | 7a | 73 | 1,616 | 9,543 | 52.7 | 21333 | **13361** |
| *Phytophthora rubi* | 7 | 7a | 47 | 37,135 | 37,135 | 53.9 | 24938 | **15463** |
| *Phytophthora cinnamomi* | 7 | 7b | 53 | 5,777 | 5,831 | 54.0 | 17877 | **12943** |
| *Phytophthora pisi* | 7 | 7b | 58 | 8,083 | 8,083 | 54.6 | 22436 | **15495** |
| *Phytophthora cryptogea* | 8 | 8a | 63 | 19,533 | 31,280 | 51.9 | 19881 | **11876** |
|  | | | | | | | **with *Py. ultimum* var. *ultimum*** | |
| *Pythium insidiosum* | C | - | 53 | 1,192 | 2,963 | 57.0 | **19290** | |
| *Pythium oligandrum* | D | - | 36 | 724 | 727 | 53.8 | **14292** | |
|  | | | | | | | **with *Plasmopara halstedii*** | |
| *Plasmopara viticola* | - | - | 75 | 1,883 | 3,995 | 43.1 | **12048** | |
|  | | | | | | | **in conjunction with GeneMark-ES** | |
| *Pilasporangium apinafurcum* | - | - | 37 | 280 | 855 | 43.9 | **13511** | |
