# Supplementary material for: Phylogenomic Reconstruction of the Oomycete Phylogeny Derived from 37 Genomes
Source: mSphere. 2017 Apr 12;2(2):e00095-17. doi: 10.1128/mSphere.00095-17 (PMC5390094; doi:10.1128/mSphere.00095-17)
Supplement: TABLE S2 [file sph002172267st5.docx]

**Table S2**

| **Single-copy phylogenies (2,280 in total)** | | **Multi-copy phylogenies (6,055 in total)** | |
| --- | --- | --- | --- |
| **Model** | **# of families** | **Model** | **# of families** |
| JTT | 898 | LG | 2859 |
| LG | 746 | JTT | 1676 |
| WAG | 307 | WAG | 708 |
| Dayhoff | 82 | Dayhoff | 198 |
| HIVb | 74 | HIVb | 121 |
| FLU | 29 | CpREV | 104 |
| CpREV | 28 | FLU | 83 |
| DCMut | 27 | RtREV | 79 |
| VT | 26 | MtArt | 55 |
| Blosum62 | 19 | VT | 52 |
| RtREV | 17 | DCMut | 46 |
| MtArt | 15 | Blosum62 | 39 |
| MtREV | 7 | MtREV | 18 |
| MtMam | 4 | MtMam | 13 |
| HIWw | 1 | HIWw | 4 |
