# Supplementary figures and images for: Phylogenomic Reconstruction of the Oomycete Phylogeny Derived from 37 Genomes
Source: mSphere. 2017 Apr 12;2(2):e00095-17. doi: 10.1128/mSphere.00095-17 (PMC5390094; doi:10.1128/mSphere.00095-17)

0.1

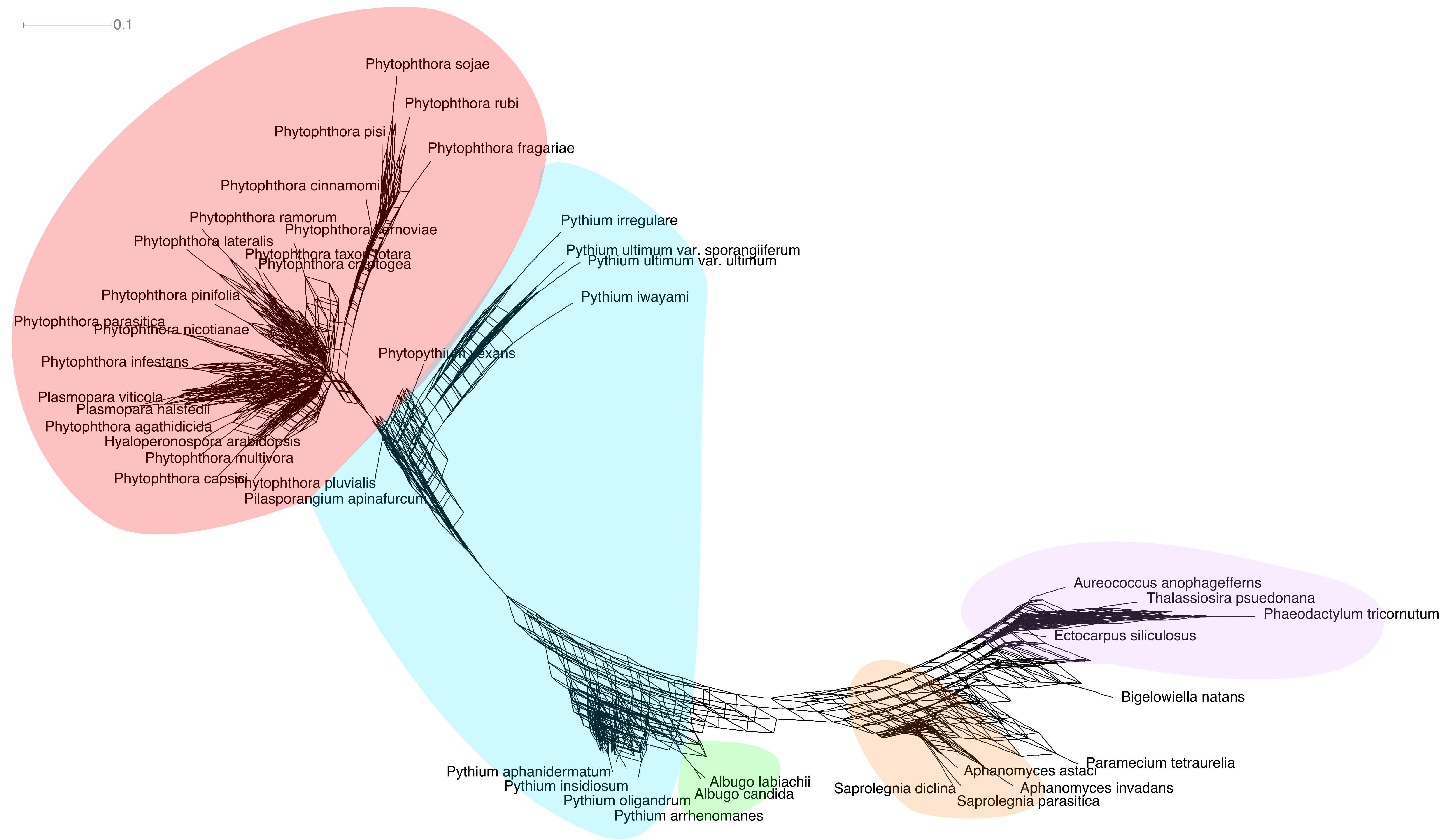

Supplement: FIG S1 [file sph002172267sf1.pdf]

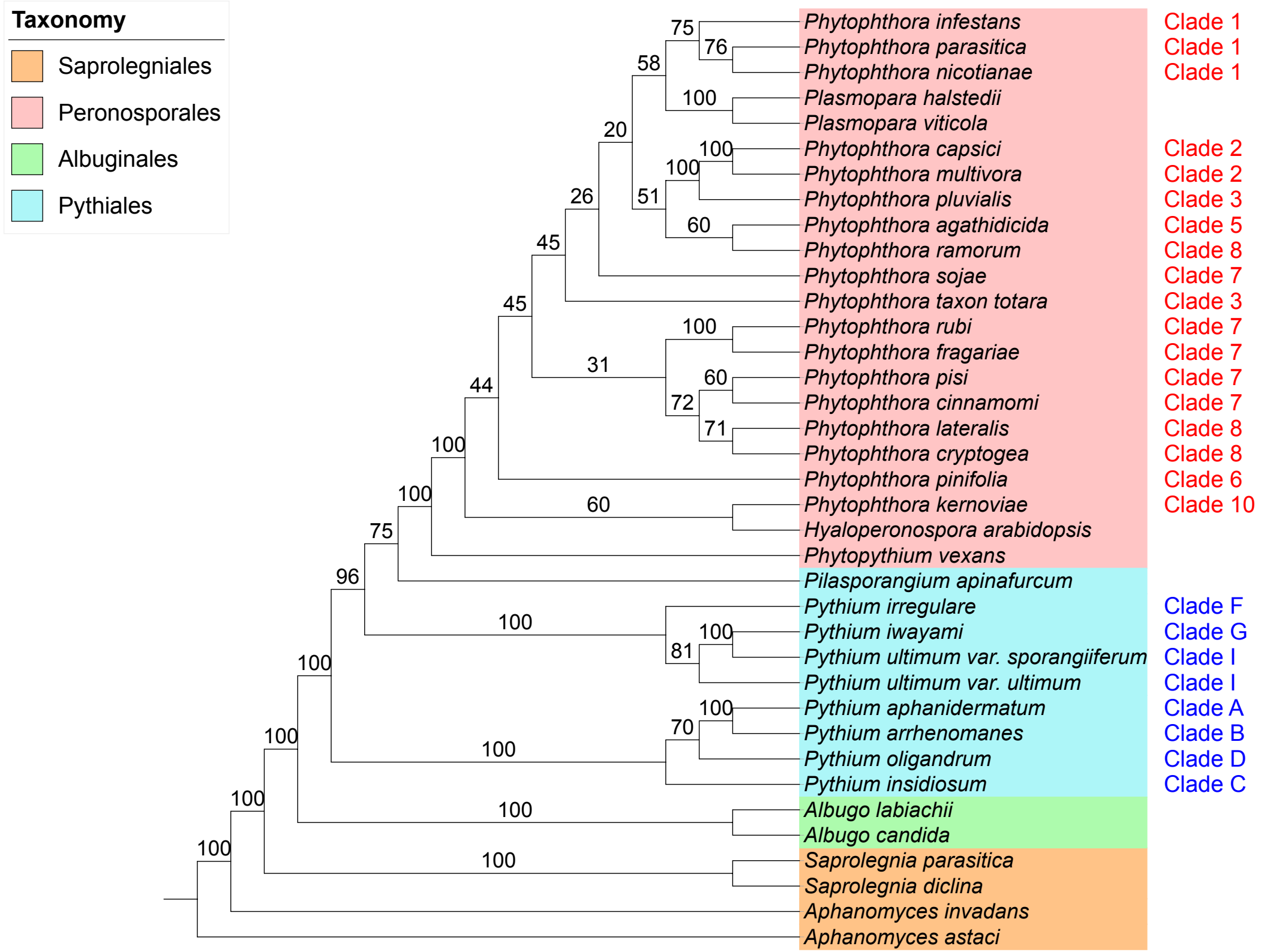

Supplement: FIG S2 [file sph002172267sf2.pdf]

0.01

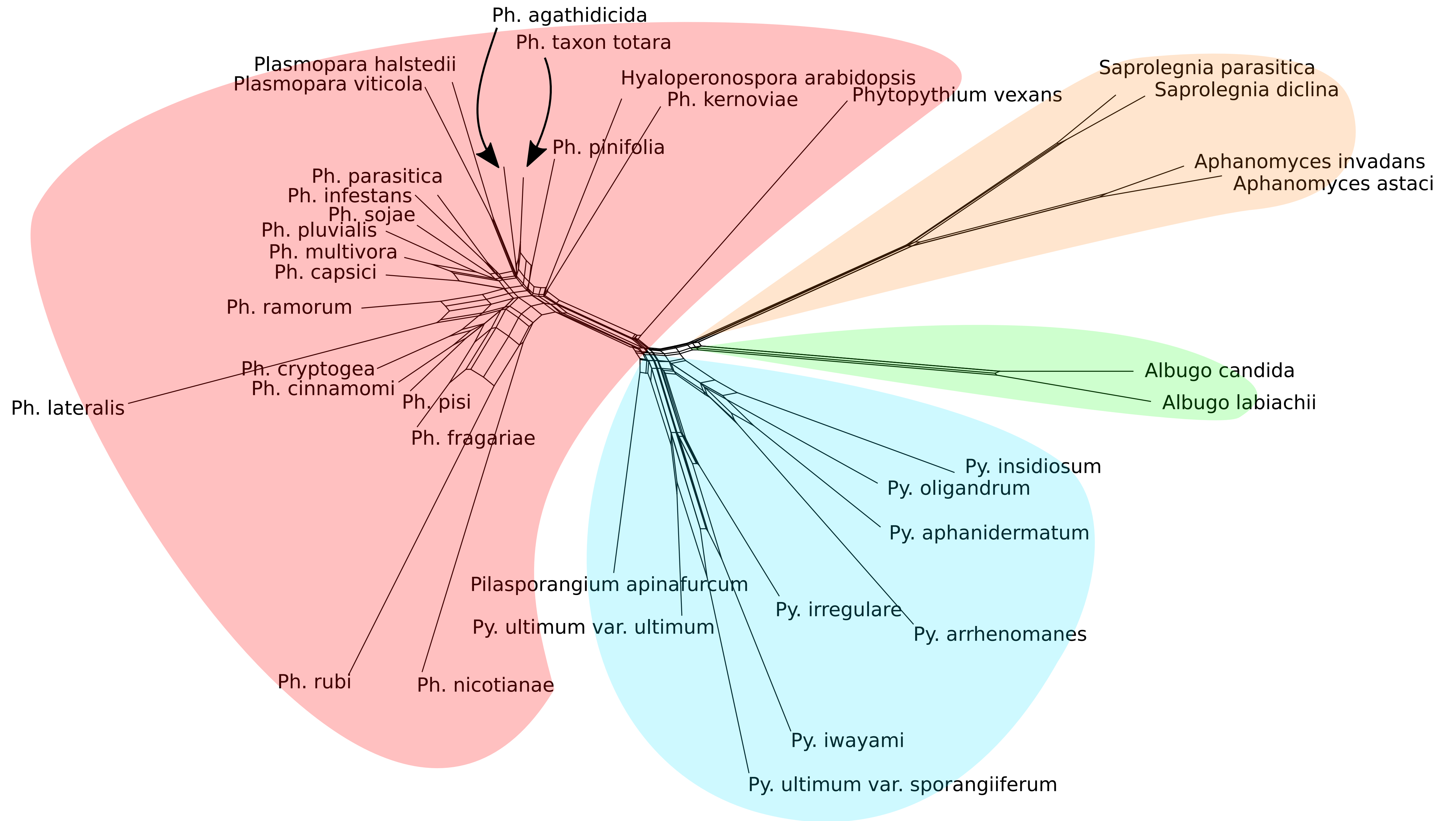

Supplement: FIG S3 [file sph002172267sf3.pdf]
